# New Perspective for Soft Tissue Closure in Medication-Related Osteonecrosis of the Jaw (MRONJ) Using Barbed Sutures

**DOI:** 10.3390/jcm10081677

**Published:** 2021-04-14

**Authors:** Johannes Laimer, Martin Hechenberger, Johanna Maria Lercher, Eva Born, Michael Schomaker, Sibylle Puntscher, Uwe Siebert, Emanuel Bruckmoser

**Affiliations:** 1 University Hospital for Craniomaxillofacial and Oral Surgery, Medical University Innsbruck, A-6020 Innsbruck, Austria; johannes.laimer@i-med.ac.at (J.L.); ddr.hechenberger@gmail.com (M.H.); johanna.lercher@student.i-med.ac.at (J.M.L.); evaborn@posteo.de (E.B.); 2 Institute of Public Health, Medical Decision Making and Health Technology Assessment, Department of Public Health, Health Services Research and Health Technology Assessment, UMIT—University for Health Sciences, Medical Informatics and Technology, A-6060 Hall in Tirol, Austria; michael.schomaker@umit.at (M.S.); sibylle.puntscher@umit.at (S.P.); uwe.siebert@umit.at (U.S.); 3 Centre for Infectious Disease Epidemiology and Research, University of Cape Town, 7550 Cape Town, South Africa; 4 Center for Health Decision Science, Departments of Epidemiology and Health Policy & Management, T.H. Chan School of Public Health, Harvard University, Boston, MA 02115, USA; 5 Institute for Technology Assessment and Department of Radiology, Massachusetts General Hospital, Harvard Medical School, Boston, MA 02114, USA; 6 Private Practice for Oral and Maxillofacial Surgery, A-5020 Salzburg, Austria

**Keywords:** medication-related osteonecrosis of the jaw (MRONJ), bisphosphonate, denosumab, exposed necrotic jaw bone, soft tissue closure, barbed suture, surgical, treatment

## Abstract

The aim of this study was to compare the effectiveness of barbed versus smooth sutures for soft tissue closure of exposed jawbone sites in medication-related osteonecrosis of the jaw (MRONJ) patients. Exposed necrotic jawbone sites surgically managed by intraoral soft tissue closure were evaluated. Either barbed sutures (Stratafix™ or V-Loc™) together with Prolene^®^ or Vicryl^®^ sutures were used. We estimated the effect of barbed sutures (BS) with Prolene^®^ compared to smooth sutures (Vicryl^®^) on the hazard rate of intraoral soft tissue dehiscence using a multivariate Cox regression model within a target trial framework, adjusting for relevant confounders. In total, 306 operations were performed in 188 sites. In the primary analysis 182 sites without prior surgery were included. Of these, 113 sites developed a dehiscence during follow-up. 84 sites were operated using BS and Prolene^®^. A total of 222 sites were operated with Vicryl^®^ (control group). In the BS group, the median time to event (i.e., dehiscence) was 148 days (interquartile range (IQR), 42–449 days) compared to 15 days (IQR, 12–52 days) in the control group. The hazard rate of developing intraoral dehiscence was 0.03 times (95%-confidence interval (CI): 0.01; 0.14, *p* < 0.001) lower for BS patients compared to the control group. Within the limits of a retrospective study, BS showed a high success rate and are therefore recommended for soft tissue closure of exposed jawbone sites in MRONJ patients. Additional studies are warranted to further evaluate this novel application of BS.

## 1. Introduction

### 1.1. Medication-Related Osteonecrosis of the Jaw (MRONJ)

Medication-related osteonecrosis of the jaw (MRONJ) is a typical side effect of antiresorptive drugs, which are for example used in osteoporosis or tumor patients with osseous metastases. Areas of exposed necrotic jaw bone, pain, infection, and a high rate of recurring dehiscences following surgical soft tissue closure dominate the clinical picture [1]. The risk of developing MRONJ lies between 0.01% and 0.03% in osteoporotic, and between 1.3% and 1.8% in oncologic patients [2].

The first report of MRONJ was published in 2003 and, at the time, was referred to as BRONJ (bisphosphonate-related osteonecrosis of the jaw) because it was first seen as a side effect in patients receiving bisphosphonate therapy for osteoporosis or bone metastases [3,4]. Several years later, the same disease pattern was observed in patients who received the monoclonal antibody denosumab [5]. Recently, also antiangiogenetic drugs and tyrosine kinase inhibitors have been reported to cause this type of jaw osteonecrosis [6]. Therefore, this adverse effect is now referred to as medication-related osteonecrosis of the jaw (MRONJ) to account for the considerable variety of causative drugs [7].

According to the latest position paper of the American Association of Oral and Maxillofacial Surgeons [1], MRONJ is characterized by exposed bone or bone that can be probed through an intraoral or extraoral fistula(e) in the maxillofacial region that has persisted for more than eight weeks in subjects treated with antiresorptive or antiangiogenic agents who do not have any history of radiation therapy to the jaws or obvious jaw metastases. MRONJ can be classified into four stages. In stage 0, there is no clinical evidence of necrotic bone. However, patients present non-specific symptoms or clinical and radiographic findings including alveolar bone loss or resorption not attributable to chronic periodontal disease, changes to trabecular pattern, regions of osteosclerosis involving the alveolar bone, or thickening of the alveolar lamina dura with decreased size of the periodontal ligament space. Stage 1 is characterized by exposed necrotic bone or fistulae with probing to bone. However, patients are asymptomatic without any signs of infection. In stage 2, exposed necrotic bone is associated with infection as evidenced by pain (with or without purulent drainage). In addition to these symptoms and signs, stage 3 patients suffer from complications, such as pathologic fracture, extra-oral fistula, oro-antral/oro-nasal communication, and others.

Regarding MRONJ treatment, there is still no internationally accepted consensus to date [8]. Conservative approaches comprise of improvement and maintenance of good oral hygiene, elimination of active dentoalveolar pathologies, and application of antibacterial mouth rinses and systemic antibiotic therapy [9]. Surgical management includes sequestrectomy, surgical debridement, and jaw osteotomies with an overall success rate of at least 58% [10]. In a multicenter case registry study evaluating treatment outcomes in patients with advanced cancer, the success rate (MRONJ resolution) for the “medication and surgery” protocol was 41.2% [11]. With the same strategy, disease improvement was achieved in 21.6%. Regarding aggressive surgery, low cumulative recurrence rates of 3.1% and 9.4% at 3 and 6 months, respectively, in resected jaws were reported [12]. Comparison between studies is often difficult since well-documented reports are scarce due to the lack of well-established protocols and significant differences regarding sample size, surgical treatment modalities, and outcomes assessed [13]. Overall, surgical treatment of jaw osteonecrosis in oncologic patients remains challenging [14]. 

### 1.2. Barbed Sutures (BSs)

Over the past decades, considerable progress has been made in the field of surgical suture materials, for example with synthetic polymers degrading in a commensurate fashion and with improvements regarding sterilization procedures [15]. One of the crucial key elements for successful wound closure is to approximate but not “strangulate” the wound edges to avoid ischemia and wound dehiscence. This is, however, an inherent risk of a traditional suture based on constricting loops [15]. 

Sutures can be categorized according to a variety of features [16]. Regarding physical characteristics, stiffness/flexibility, degradation properties (absorbable versus non-absorbable), and tensile strength (knot-pull versus straight-pull strength) can be distinguished. With regard to structural characteristics, there are suture size, filament structure (multifilament, monofilament, and pseudo-monofilament), and surface texture (smooth versus barbed) to be distinguished. 

**Figure 1:**
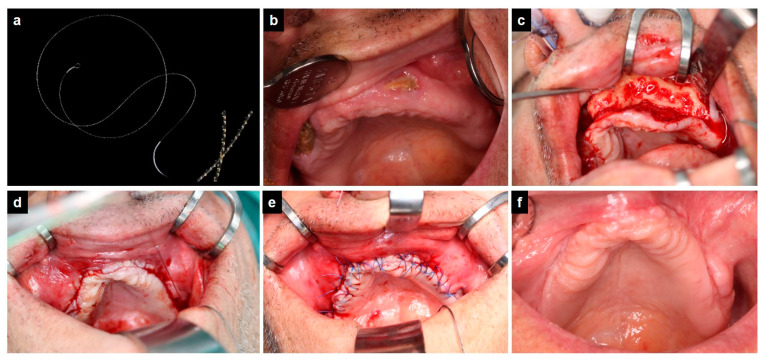
Intraoral soft tissue closure of maxillary mucosal dehiscence. (**a**): barbed suture with a magnified cutout in the right bottom corner showing details of barbs. (**b**): Preoperative picture of two maxillary sites with exposed necrotic jaw bone, (**c**): intraoperative image showing mucoperiostal flap elevation, (**d**): intraoperative image showing soft tissue closure with BS, (**e**): intraoperative image showing final situation after superficial suturing with Prolene^®^, and (**f**): follow-up image demonstrating completely healed intraoral mucosa without any dehiscence.

Barbed sutures (BSs) belong to the category of straight-pull strength sutures and do not require constricting loops to keep the wound edges together. They have sharp projections or barbs on their surface to help anchor the suture to the tissue linearly [16]. For deep wound closures, multifilament barbed sutures (“intertwined”) with improved mechanical properties (more flexibility and pliability) are ideal. The flexibility of the barb depends on both barb geometry and design, which need to be adapted for use in different types of tissue [17]. An example of a BS is depicted in Figure 1a. The suture includes an anchoring loop at the end, which is opposed to the end with the needle. After the first bite, the needle is passed through this loop for fixation of the suture at the point where the surgeon starts the procedure. The barbs are depicted in detail in the bottom right corner of Figure 1a. These directional projections (barbs) provide multiple anchoring points enabling a knotless suturing technique.

Barbed sutures are currently used in various surgical fields one of which is plastic surgery. Applications include aesthetic breast surgery [18], correction of protruding ears [19], body contouring surgery [20], “lunch time” lifting [21], or reduction of face and neck laxity [22]. Apart from these esthetic indications, barbed sutures have also been used for pharyngoplasty [23], joint arthroplasty [24,25], tendon repair [26,27], minimally invasive gynecologic surgery [28,29], minimally-invasive prostatectomy [30,31], and in general and digestive surgery [32]. To date, the comparative effectiveness of BS in oral surgery has not been reported in the international literature.

### 1.3. Study Aim

The purpose of this retrospective study was to assess the comparative effectiveness of barbed versus smooth sutures for closure of exposed necrotic jawbone sites in patients suffering from MRONJ. Since the most frequent and challenging setback for this type of surgery is postoperative wound dehiscence, we hypothesized that a straight-pull strength suture allowing for perfectly tight soft tissue closure could potentially improve outcomes and lower the respective dehiscence rate.

## 2. Materials and Methods

### 2.1. Study Design

In this retrospective study, we collected data from MRONJ patients who had undergone at least one operation for intraoral soft tissue closure at the University Hospital for Craniomaxillofacial and Oral Surgery Innsbruck, Austria. The study was approved by the institutional board in charge, that is, the ethics committee of the Medical University Innsbruck, Austria (reference number 1064/2020). The study was conducted in full accordance with the principles expressed in the Declaration of Helsinki, revised in 2013.

Subjects were included up to December 2019. Until the end of August 2016, only synthetic absorbable braided 4-0 sutures (Vicryl^®^, Ethicon, Cincinnati, OH, USA) were used for intraoral soft tissue closure in areas of the exposed necrotic jaw bone. The suturing technique included horizontal mattress sutures, back stitches, and single knots for superficial closure of the intraoral mucosa. Although resorbable, these sutures were removed around 10–14 days postoperatively for evaluation purposes and to facilitate oral hygiene. All sites of exposed necrotic jaw bone, which were operated solely using Vicryl^®^ sutures served as the control group for the BS collective.

Along with the establishment of a specialized MRONJ clinic in 2016, more and more patients have been operated with running intramucosal absorbable monofilament unidirectional BS. These sutures were combined with superficial monofilament non-absorbable 4-0 sutures (Prolene^®^, Ethicon, Cincinnati, OH, USA) in the horizontal mattress and single knot technique. BS used were either Stratafix™ 4-0 (Ethicon, Cincinnati, OH, USA) or V-Loc™ 4-0 (Covidien, Minneapolis, MN, USA) depending on the availability of these materials. 

Irrespective of the suture material applied, resection of necrotic jawbone was performed until bleeding of viable bone was obtained. This was followed by smoothening of sharp bone edges and subsequent soft tissue closure (mucoperiostal flap) using either BS and Prolene^®^ 4-0 or Vicryl^®^ 4-0 sutures as described above. A clinical example of a typical surgery using BS is shown in Figure 1 and in the Supplementary Video S1.

The suturing technique includes the following steps. After preparation of a tension-free mucoperiostal flap, a deep bite in the muscle or periostal layer at one end of the wound is taken. Then, the needle is passed through the preformed anchor loop for secure fixation. The tissue is approximated using a continuous suture path in a running suture like manner. The needle enters the tissue in the periosteal layer close to the wound edge and must exit right at the cutting edge of the mucosa for optimum apposition of wound edges. Gentle traction is to be applied in order to approximate the edges and close the wound. At the end of the procedure, a back stitch is taken in the reverse direction away from the apex of the wound, and the needle is guided to exit through the mucosa about 1–2 cm lateral to the end of the wound to lock the stitch. Gentle traction on the suture further optimizes the wound closure. Finally, the suture is cut flush with the mucosa.

Regarding the antibiotic regimen, mostly a combination of ampicillin and sulbactam or clavulanic acid was used. Patients allergic to penicillin were given clindamycin. Antibiotics were started a few days before surgery (median, 3 days) and were continued for several days postoperatively (median for intravenous antibiotics, 6 days; median for oral antibiotics, 5 days).

We included surgically managed MRONJ patients with a history of antiresorptive therapy (bisphosphonates and/or denosumab) who met the MRONJ criteria as published by the American Association of Oral and Maxillofacial Surgery in 2014 [1]. MRONJ stages one to three based on this classification were eligible for inclusion. We excluded patients with mandibular fractures, subjects having undergone partial mandibular resection with reconstruction using a titanium plate, and cases of free flap reconstructions.

Follow-up visits included a thorough intraoral examination to determine surgical success or failure. The latter was defined as jaw bone, which could be probed with a standard perio probe, persistence of a fistula, or visible dehiscence of the intraoral mucosa with gaping wound margins and exposed bone. Along with the establishment of our specialized MRONJ clinic as mentioned above, a stricter schedule for postoperative follow-ups was introduced. Visits were then regularly scheduled at week 2 after surgery for removal of the Prolene^®^ sutures, at week 4 and 8, and thereafter every three to six months depending on the individual situation. 

### 2.2. Target Trial

In order to avoid immortal time bias, confounding, and selection bias, we followed the modern causal approach of emulating a (hypothetical) randomized controlled target trial using our observational data [33,34]. We emulated the following target trial [33,34]: eligible were patients with a history of antiresorptive therapy, MRONJ stages one to three (except for stage three patients with mandibular fractures, partial mandibular resection with titanium plate reconstruction, and free flap cases) who underwent intraoral soft tissue closure and had no prior surgery of the respective site. Treatment strategies comprised BS (Stratafix™ or V-Loc™) and Prolene^®^ versus Vicryl^®^. Individuals were not randomly assigned to the BS and control group, but rather operated based on availability at the respective date and surgeon’s preference. Patients were followed while in care (from the day of first operation), and censored at study-end-date (December 2019) or when wound dehiscence was diagnosed, whatever occurred first. Under the assumption that date and surgeon would be the only variables affecting both the choice of suture material and development of dehiscence, and surgical procedures were performed in the same manner, the causal effect [35] of suture type on dehiscence risk can be estimated using a Cox proportional hazards model, including both date and surgeon as covariates [36].

### 2.3. Statistical Analysis

The patient population is described using means, standard deviations, proportions, medians, and interquartile ranges (IQRs), as appropriate and stratified by suture type. The crude (unadjusted) risk of developing post-operative wound dehiscence was estimated using Kaplan–Meier curves, comparing patients with suture type including BS (Stratafix™ or V-Loc™) and Prolene^®^ versus patients with Vicryl^®^ (control group). We used a multivariate Cox proportional hazard model to estimate the hazard ratio (HR) with 95% confidence interval (95%-CI) for the time to dehiscence and to adjust for potential confounding. The proportional hazards assumption was checked by inspection of log–log-plots. If patients had more than one surgery, the dependence between the respective surgeries was incorporated into the model using frailties where possible. Covariate inclusion was guided by causal principles [35], hence, date and surgeon were included as confounders. Date was included linearly as a continuous variable measured in number of days from first study date. Clinician was included as a categorical variable, where surgeons having performed <15 operations were grouped together. In the emulated target trial, individuals would be randomly assigned to one of the two groups because of the inclusion of the measured confounders and under the assumptions outlined above. In sensitivity analyses, we used Cox models with interactions to explore whether the treatment effect varies with respect to biphosphonate and denosumab exposure, advanced MRONJ stage and cancer patients.

## 3. Results

From December 2006 to December 2019, a total of 306 operations were performed in 188 sites (areas of exposed necrotic jawbone) in 150 MRONJ patients. The surgeries also included reoperations of sites, which became dehiscent following primary attempts of intraoral soft tissue closure. In 41 patients 124 sites were excluded from the primary analysis, as they did not conform with the target trial protocol due to prior surgery, but were included in the secondary analysis. This left 182 sites in 108 patients for the primary, and 306 sites for the secondary analysis.

Of the included sites 113 developed a dehiscence during follow-up. Starting in August 2016, 84 sites in 55 patients were operated using BS and Prolene^®^. In 100 patients 222 sites were operated with Vicryl^®^ 4-0. The last operation using Vicryl^®^ was done in October 2018. Overall median time to event was 29 days (IQR, 13–177 days). Patients of the BS group had a median time to event of 148 days (IQR, 42–449 days) compared to 15 days (IQR, 12–52 days) in the control group. Mean and median follow-up time were 280.5 and 122 days (range, 3–2082 days), respectively. Basic descriptive data of the study population are summarized in Table 1.

**Figure 2:**
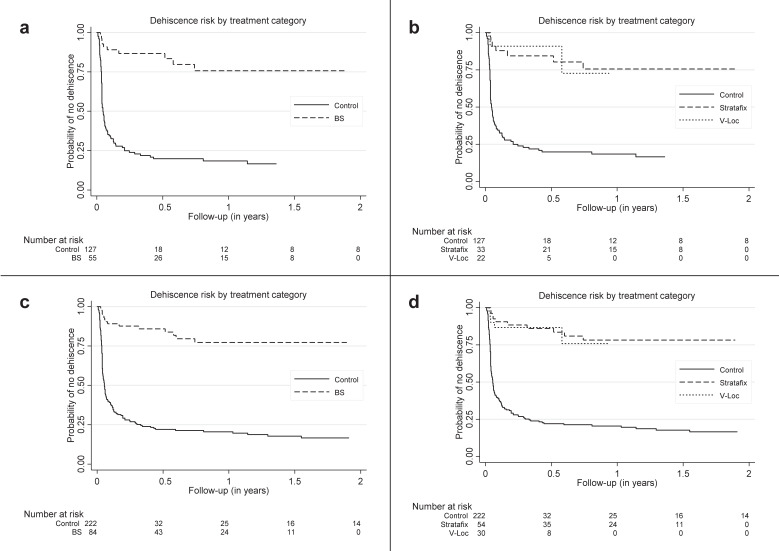
Kaplan–Meier plots of treatment groups for all patients and for patients without prior surgery. (**a**): barbed sutures (BS) and control group including patients without prior surgery; (**b**): Stratafix, V-Loc™, and control group including patients without prior surgery; (**c**): BS and control group including all patients; and (**d**): Stratafix, V-Loc™, and control group including all patients.

Comparing both groups, a much larger proportion of patients in the BS group stayed without dehiscence compared to the control group (Figure 2), during follow-up. The differences between the Stratafix™ and the V-Loc™ group were small. The adjusted hazard rate of developing dehiscence was 0.03 times (95%-CI: 0.01; 0.14, *p* < 0.001) smaller for BS patients compared to the control group. Our sensitivity analyses showed that this main result remained similar if no covariate adjustment was performed (HR: 0.13, 95%-CI: 0.06; 0.25, *p* < 0.001), or if the analysis was extended to patients with prior surgery (HR: 0.08, 95%-CI: 0.03; 0.21, *p* < 0.001), or for cancer patients (HR: 0.04, 95%-CI: 0.01; 0.17, *p* < 0.001). Details are shown in Table 2. Patients with a higher MRONJ stage had a slightly higher risk. Similarly, patients treated with biphosphonates and denosumab in succession had a slightly higher risk than patients receiving either biphosphonates or denosumab alone.

Subgroup analysis revealed that a collective of twelve patients who had primarily been operated with Vicryl^®^ and who developed a dehiscence postoperatively, were then successfully managed using BS. The median time from surgery to dehiscence in this subgroup was 14 days for Vicryl^®^ (range, 13–98 days) and 333 days for BS (range, 141–480 days). This difference was statistically significant (*p*-value: 0.013). All of the respective twelve patients were reoperated using BS. Two of these patients developed a dehiscence following surgery with BS. However, these two patients were reoperated with BS again and did not show any evidence of intraoral dehiscence at the study end date.

## 4. Discussion

The key result of our study is that the success rate of soft tissue closure in MRONJ patients using BS and Prolene^®^ is clinically and statistically significantly higher compared to soft tissue closure using Vicryl^®^ 4–0. This outcome signifies a major advance in this drug-induced condition since postoperative wound dehiscence still represents the main drawback following such surgical interventions.

Although the risk to develop MRONJ up to 1.8% in oncologic patients may seem relatively small, this drug side effect represents a potentially severe and quality of life impairing condition. With the broad use of highly potent bisphosphonates (particularly zoledronic acid) and the monoclonal antibody denosumab in recent years, MRONJ has become a serious interdisciplinary problem on a worldwide scale.

To date, no international consensus regarding a reliable treatment to provide both minimal invasiveness and a high success rate has been obtained. Conservative therapy primarily aims at alleviation of symptoms and signs of the disease without basically solving the problem. On the other end of the therapeutic spectrum, complete resection of all necrotic jaw bone followed by free flap reconstruction claims to solve the problem at the base. However, this approach quite obviously implies maximum invasiveness, which has to be taken into consideration regarding the typical MRONJ collective of elderly tumor patients in palliative care.

A less invasive approach is described in several papers evaluating surgery for intraoral soft tissue closure. In a retrospective cohort study, double-layer closure techniques including mylohyoid muscle flaps and pedicled buccal fat flaps have been evaluated [37]. Mucosal integrity at the last follow-up was reported in 88.0% (44 of 50) of patients in the mylohyoid muscle flap group and 93.1% (27 of 29) of patients in the pedicled buccal fat flap group. In a prospective study only including MRONJ stage III patients with a minimum follow-up of 6 months, different soft tissue closure techniques (mylohyoid muscle flap, buccal fat flap, or mucoperiosteal flap alone) were evaluated. In 12 of the 44 cases, relapses occurred [38]. Better results regarding soft tissue healing and recurrence rate were achieved with the muscle or fat flap. In 18 cases, partial hypoesthesia of the lip was noted. Overall, 38 patients reached mucosal integrity within the follow-up.

There is still need for a minimally invasive therapy with a high success rate. We considered the treatment approach proposed in our study as minimally invasive since it does not include extensive bone resection with free flap reconstruction. The success rate can be regarded as remarkable, especially when compared to the high dehiscence rate following soft tissue closure with conventional smooth sutures. From our experience, we assume that most colleagues treating MRONJ patients would often be frustrated by the high dehiscence rate short time after surgery. We even hypothesized a publication bias and speculate that the overall dehiscence rates reported in the international literature may be much higher since negative treatment results tend to be published less likely than positive outcomes.

Of particular interest appears the subgroup of twelve patients who developed dehiscences following surgery with Vicryl^®^ 4-0. All of these patients were successfully managed using BS and Prolene^®^ (two of the subjects had to be reoperated twice). In all cases, suspicious areas of jaw bone indicative of osteonecrosis were removed, and bony spurs and edges were recontoured during the reoperation. In these twelve patients, the novel treatment approach with BS has been a significant advance as it was demonstrated that even complex cases of intraoral dehiscence could be successfully managed with BS. Based on the operations performed over the last years, we assumed that the perfectly tight wound closure achieved with BS is probably the key factor for a stable result. This impressive quality of wound closure can be seen in Figure 1d. Consequently, we encourage surgeons to go for another attempt of intraoral soft tissue closure using BS considering the high success rate of this technique as compared to approaches using conventional suture material.

This study had several limitations. First, this study was lacking a standardized follow-up protocol in the Vicryl^®^ group (apart from suture removal 10–14 days postoperatively) because a clear schedule for control visits was only established after initiation of our special MRONJ outpatient clinic. However, it can be assumed that patients developing dehiscence would mostly return to clinic because of the considerable complaints, which this condition can cause. This reduced the likelihood of losses to follow-up.

Second, as all observational studies and comparison to historical controls, our study has the potential of confounding because variables cannot be controlled in a retrospective design. However, under the assumption that date and surgeon would be the only variables affecting both the choice of suture material and the outcome dehiscence, and otherwise, the surgical procedures were performed in the same standardized manner, our analysis based on target trial framework likely provides a valid causal effect [36,39,40]. Our knowledge on how the surgeries were performed suggests that these assumptions may be met, and therefore further confounding is unlikely. In addition, considering the strong effect of a HR of 0.03 indicating a high success rate when using BS, a prospective randomized trial could be problematic. However, if justifiable from an ethical point of view, a randomized controlled trial would provide the best evidence possible.

Third, the heterogeneity of participants in our study limits generalizability. Some patients had undergone prior surgery, there were several surgeons involved, and MRONJ stage, causative drug type, and underlying disease differed between subjects. In the BS group, two different types of sutures (Stratafix™ and V-Loc™) were used. However, all of these potential effect modifiers were statistically analyzed, and the results remained statistically significant in our sensitivity analyses.

Finally, it should be noted that BS were compared to Vicryl^®^ 4-0 only. In other words, the effectiveness in the control group may have been better if a different suturing material had been used, for example a nonresorbable monofilament type of material. However, Vicryl^®^ is undoubtedly one of the most commonly used materials worldwide and therefore appears to be a suitable candidate for comparison.

## 5. Conclusions

We reported considerable progress and significant advance for intraoral soft tissue closure in MRONJ patients achieved by use of BS and Prolene^®^ instead of conventional smooth suture material (Vicryl^®^). The respective success rate was remarkable, taking into account potential confounders and effect modifiers such as a surgeon, underlying disease, drug type, BS type, or MRONJ stage. In light of these promising clinical results, we advocated the use of BS for intraoral soft tissue closure in MRONJ patients with areas of exposed jawbone. However, inherent limitations due to the retrospective study design must be taken into consideration. Additional studies are warranted to further evaluate this novel application of BS. If ethically justifiable, a randomized controlled trial could provide the best scientific evidence in this regard. Future research should also be performed to evaluate the potential benefit of BS (not relying on constricting loops) in alveolar ridge augmentation, since a tension-free wound closure is essential for such procedures to avoid postoperative dehiscence eventually leading to partial or total failure and loss of the augmentation material. 

## Figures and Tables

**Table 1:** Basic characteristics and outcomes of study and control group with and without prior surgery.

		Patients without Prior Surgery		Total	All Patients		Total
		BS Group *n* (%)	Control Group *n* (%)		BS Group *n* (%)	Control Group *n* (%)	
Outcome	Dehiscence yes	10 (18.2%)	103 (81.1%)	113 (62.1%)	15 (17.9%)	177 (79.7%)	192 (62.7%)
	Dehiscence no	45 (81.8%)	24 (18.9%)	69 (37.9%)	69 (82.1%)	45 (20.3%)	114 (37.3%)
Time to event in days	Mean (SD)	234.76 (219.94)	117.04 (278.19)	152.62 (266.92)	243.17 (210.88)	128.97 (300.29)	160.32 (282.93)
	Median IQR (25–75%)	148 407 (42–449)	15 40 (12–52)	29 164 (13–177)	195.5 398.5 (48–446.5)	19 54 (12–66)	30 168 (14–182)
Surgeons	1	30 (54.6%)	5 (3.9%)	35 (19.2%)	44 (52.4%)	9 (4.0%)	53 (17.3%)
	2	17 (30.9%)	0 (0%)	17 (9.3%)	28 (33.3%)	3 (1.4%)	31 (10.1%)
	3	7 (12.7%)	6 (4.7%)	13 (7.1%)	9 (10.7%)	7 (3.2%)	16 (5.2%)
	Other	1 (1.8%)	116 (91.4%)	117 (64.3%)	3 (3.6%)	203 (91.4%)	206 (67.3%)
Stage	1	17 (30.9%)	20 (15.8%)	37 (20.3%)	32 (38.1%)	58 (26.1%)	90 (29.4%)
	2	30 (54.5%)	94 (74.0%)	124 (68.1%)	37 (44.0%)	137 (61.7%)	174 (56.9%)
	3 (fistulas)	8 (14.6%)	13 (10.2%)	21 (11.5%)	15 (17.9%)	27 (12.2%)	42 (13.7%)
Drugs	Biphosphonate only	8 (14.6%)	57 (44.9%)	65 (35.7%)	15 (17.9%)	106 (47.8%)	121 (39.5%)
	Denosumab only	29 (52.7%)	42 (33.1%)	71 (39.0%)	40 (47.6%)	61 (27.5%)	101 (33.0%)
	Biphosphonate -> Denosumab	13 (23.6%)	24 (18.9%)	37 (20.3%)	21 (25.0%)	48 (21.2%)	69 (22.5%)
Cancer	Yes	47 (85.4%)	111 (87.4%)	158 (86.8%)	72 (85.7%)	189 (85.1%)	261 (85.3%)
	No	8 (14.6%)	16 (12.6%)	24 (13.2%)	12 (14.3%)	33 (14.9%)	45 (14.7%)
Sex	Men	20 (36.4%)	52 (40.9%)	72 (39.6%)	27 (32.1%)	83 (37.4%)	110 (35.9%)
	Women	35 (63.6%)	75 (59.1%)	110 (60.4%)	57 (67.9%)	139 (62.6%)	196 (64.1%)
Age at surgery in years	Mean (SD)	69.2 (11.7)	68.8 (12.6)	68.9 (12.3)	69.5 (12.1)	68.6 (13.2)	68.9 (12.9)
	Median IQR (25–75%)	71.9 18.7 (59.7–78.4)	72.6 17.7 (60.9–78.6)	72.5 18.1 (60.5–78.6)	72.7 19.1 (60.1–79.2)	73.4 19.6 (59.5–79.1)	73.2 19.4 (59.7–79.1)

BSs: barbed sutures, SD: standard deviation, IQR: interquartile range.

**Table 2:** Crude and adjusted hazard ratios including 95% confidence intervals and *p*-values for the BS and control group.

Setting	Hazard Ratio (HR) BS versus Vicryl^®^	95% CI	*p*-Value
Patients without prior surgery, crude effect	0.13	0.06; 0.25	<0.001
Patients without prior surgery, adjusted for surgeon and calendar time	0.03	0.01; 0.14	<0.001
Patients without prior surgery, crude effect, for three treatment groups	0.14 (Stratafix™) 0.11 (V-Loc™) Ref (Vicryl^®^)	0.07; 0.31 0.04; 0.35	<0.001 <0.001
Patients without prior surgery, adjusted for surgeon and calendar time, for three treatment groups	0.04 (Stratafix™) ^a^ 0.03 (V-Loc™) ^a^ Ref (Vicryl^®^)	0.01; 0.16 0.01; 0.15	<0.001 <0.001
All patients, crude effect	0.14 ^a^	0.08; 0.23	<0.001
All patients, adjusted for surgeon and calendar time	0.08 ^a^	0.03; 0.21	<0.001
Patients without prior surgery, crude effect, effect modification of stage	0.20 (stage 1) ^a^ 0.14 (stage 2) ^a^ 0.06 (stage 3) ^a^	0.06; 0.69 0.06; 0.32 0.01; 0.48	0.011 <0.001 0.008
Patients without prior surgery, adjusted for surgeon and calendar time, effect modification of stage	0.05 (stage 1) ^a^ 0.04 (stage 2) ^a^ 0.02 (stage 3) ^a^	0.01; 0.30 0.01; 0.16 0.01; 0.19	0.001 <0.001 0.001
Patients without prior surgery, crude effect, effect modification of cancer	0.16 (cancer) ^a^	0.08; 0.30	<0.001
Patients without prior surgery, adjusted for surgeon and calendar time, effect modification cancer	0.04 (cancer) ^a^	0.01; 0.17	<0.001
Patients without prior surgery, effect modification of drug types administered	0.11 (Denosumab only) ^a^ -- (Biphosphonate only) ^b^ 0.23 (Biphosphonate followed by Denosumab) ^a^	0.05; 0.26 -- 0.07; 0.80	<0.001 -- 0.020
Patients without prior surgery, adjusted for surgeon and calendar time, effect modification of drug types administered	0.06 (Denosumab only) ^a^ -- (Biphosphonate only) ^b^ 0.10 (Biphosphonate followed by Denosumab) ^a^	0.01; 0.28 -- 0.015; 0.65	<0.001 -- 0.016

BSs: barbed sutures; 95%-CI: 95% confidence interval; Main results in bold; ^a^ does not converge with frailty, calculated without frailty (i.e., dependence structure for sites that belong to the same patient); ^b^ not enough cases in each group to estimate hazard ratios.

## Data Availability

Raw data will be made available upon reasonable request.

## Supplementary Materials

The following are available online at https://www.mdpi.com/article/10.3390/jcm10081677/s1, Video S1: A clinical example of a typical surgery using BS.
